# Deposition of Aerosolized Lucinactant in Nonhuman Primates

**DOI:** 10.1089/jamp.2018.1505

**Published:** 2020-01-30

**Authors:** Timothy J. Gregory, Hammad Irshad, Ramesh Chand, Philip J. Kuehl

**Affiliations:** ^1^ Windtree Therapeutics, Inc., Warrington, Pennsylvania.; ^2^ Lovelace Biomedical, Albuquerque, New Mexico.

**Keywords:** aerosol distribution, noninvasive delivery, regional lung aerosol deposition, surfactant aerosol

## Abstract

***Background:*** Lucinactant for inhalation is an investigational noninvasive, aerosolized surfactant replacement therapy for treatment of preterm neonates with respiratory distress syndrome. Lucinactant for inhalation consists of lyophilized lucinactant and the Aerosurf^®^ Delivery System (ADS). The objective of this study was to characterize the total and regional pulmonary deposition of lucinactant delivered by the ADS in nonhuman primates (NHPs).

***Methods:*** Lucinactant was radiolabeled by the addition of technetium-99m (^99m^Tc)-sulfur colloid. The radiolabeled aerosol was characterized and validated using a Mercer cascade impactor. An *in vivo* deposition study was performed in three cynomolgus macaques. Radiolabeled lucinactant was aerosolized using the ADS and delivered via nasal cannula under 5 cm H_2_O nasal continuous positive airway pressure (nCPAP) for 5–9 minutes. A two-dimensional planar image was acquired immediately after aerosol administration, followed by a three-dimensional single-photon emission computed tomography (SPECT) image and a second planar image. The images were analyzed to determine the pulmonary (lungs) and extrapulmonary (nose + mouth, trachea, stomach) distribution. The SPECT data were used to determine regional deposition.

***Results:*** The radiolabed lucinactant aerosol had a mass median aerodynamic diameter = 2.91 μm, geometric standard deviation (GSD) = 1.81, and an activity median aerodynamic diameter = 2.92 μm, GSD = 2.06. Aerosolized lucinactant was observed to deposit in the lungs (11.4%), nose + mouth (79.9%), trachea (7.3%), and stomach (1.4%). Analysis of the SPECT image demonstrated that the regional deposition within the lung was generally homogeneous. Aerosolized lucinactant was deposited in both the central (52.8% ± 1.2%) and peripheral (47.2% ± 1.2%) regions of the lungs.

***Conclusion:*** Aerosolized lucinactant, delivered using the ADS via constant flow nCPAP, is deposited in all regions of the lungs demonstrating that surfactant can be aerosolized and delivered noninvasively to NHPs.

## Introduction

Respiratory distress syndrome (RDS) of the newborn is a disease that results from insufficiency of pulmonary surfactant in the immature neonatal lung, which carries a risk of high morbidity and mortality. Intratracheal exogenous surfactant replacement therapy (SRT) has well-established benefits in infants with RDS^(1,2)^ and has become a standard recommended therapy for this condition.^(3–6)^ Early SRT is more effective in reducing morbidity and mortality due to RDS than SRT delivered later,^(2)^ and multiple doses are sometimes necessary.^(1)^

Intratracheal instillation of surfactant into the lung requires endotracheal intubation, along with concomitant positive pressure mechanical ventilation (MV). However, endotracheal intubation is an invasive painful procedure that itself has potential deleterious effects to the infant.^(7–9)^ Furthermore, MV is associated with morbidities such as ventilator-associated lung injury, volutrauma/barotrauma resulting in pulmonary air leaks, and may also contribute to development of chronic lung disease/bronchopulmonary dysplasia (BPD).^(10)^

To avoid endotracheal intubation and MV in preterm neonates with mild-to-moderate RDS, a strategy of using nasal continuous positive airway pressure (nCPAP) as an effective means of providing ventilatory support is now an accepted practice.^(3–6,11)^ nCPAP improves respiratory function in neonates by increasing functional residual capacity, improving lung compliance, and dilating upper airway structures, thereby improving gas exchange and reducing work of breathing.^(11,12)^

Studies in very preterm neonates of initial treatment of RDS with nCPAP alone,^(13–16)^ including meta-analyses,^(17,18)^ have shown outcomes with this approach that are similar to traditional early treatment with intratracheal surfactant. Thus, the strategy of initially supporting neonates with nCPAP and reserving SRT only for those who require intubation appears to be reasonably effective and potentially safer by avoiding intubation. However, ∼33%–67% of patients required intubation and intratracheal surfactant replacement.

Several studies have demonstrated that earlier intratracheal SRT is more beneficial than later SRT.^(2)^ For this reason, guidelines recommend that when SRT is used it be given as early as possible.^(3–6)^ However, when nCPAP is used as initial respiratory support, SRT is necessarily delayed in those neonates who ultimately require endotracheal intubation.

Thus, an unmet medical need exists for a means to deliver SRT to preterm neonates with RDS supported with nCPAP early in the course of the disease. This strategy has the potential to improve RDS before the development of respiratory failure, thereby avoiding the need for endotracheal intubation and MV and the resultant potential for morbidity and complications. The ability to administer SRT via aerosol has the potential to address this unmet need.

Efforts to aerosolize surfactants in clinical models have been largely unsuccessful to date^(19)^ because of the limited capability of currently available aerosol generators to aerosolize surfactants in the amount needed to achieve a therapeutic benefit. Compared with surfactant bolus administration via endotracheal instillation, surfactant nebulization is highly inefficient and dose delivery is limited.^(20)^ Newer aerosol generator technologies may allow for administration of aerosolized surfactant in sufficient quantities to achieve a therapeutic response. As with liquid surfactant, aerosolized surfactant is most likely delivered preferentially to the ventilated parts of the lungs.^(21)^ It is therefore likely that improving lung aeration by providing appropriate ventilatory support during aerosol delivery, such as with nCPAP, would improve the delivery of aerosolized surfactant.

To address this unmet need, lucinactant for inhalation, an investigational drug-device combination product, is being developed to deliver aerosolized SRT to preterm neonates with RDS who are being supported with nCPAP. Lucinactant for inhalation is composed of a drug component, lyophilized lucinactant, and a device component, referred to as the Aerosurf^®^ Delivery System (ADS; Windtree Therapeutics, Inc., Warrington, PA). The ADS uses novel capillary-based aerosol generator technology to aerosolize lucinactant for inhalation, providing a high-density surfactant aerosol with an appropriate particle size for respiration and deposition within the neonatal lung. The ADS may allow aerosolized lucinactant to be deposited in the lungs of preterm neonates in sufficient quantities to effect a therapeutic response analogous to that of endotracheal instillation of surfactant.

The objective of this study was to characterize the total and regional pulmonary deposition of lucinactant delivered by the ADS.

## Methods

This obective was assessed through both *in vitro* and *in vivo* studies. The *in vitro* study included the development and validation of a radiolabeling procedure for lucinactant aerosolized with the ADS. The primary objectives were to: (1) adapt the prototype ADS to anesthetized nonhuman primates (NHPs), (2) radiolabel lucinactant, and (3) validate the radiolabel for delivery to NHPs.

A series of *in vivo* studies were performed with three cynomolgus macaques to evaluate the deposition of lucinactant aerosol delivered and inhaled (1) using an aerosol circuit with no continuous positive airway pressure (CPAP) applied and (2) using an nCPAP circuit.

### Lyophilized lucinactant preparation

Lyophilized lucinactant was provided in sealed vials containing 300 mg of the test article (Lot No. G15002; Windtree Therapeutics, Inc.). Sterile water for injection (WFI; NDC 0409-4887-99, Lot No. 38-454-DK; Hospira, Inc., Lake Forest, IL) was used to reconstitute the test article. Ten milliliters of WFI was added to each vial, and the vials were inverted for 60 seconds at a frequency of about 1 inversion per second. This resulted in the final concentration of 30 mg total phospholipid (TPL)/mL of the lucinactant in the vials.

### Aerosurf Delivery System

The prototype ADS was used to generate an aerosol of lyophilized lucinactant. The ADS is an investigational medical device that consists of an Aerosurf Control Unit (Serial. No. 00019; Windtree Therapeutics, Inc.) and uses Aerosurf Delivery Packs (ADP) to aerosolize the lyophilized lucinactant suspension. Three vials of reconstituted lyophilized lucinactant (30 mg TPL/mL) were added to the ADS syringe for each test. A syringe pump forces the suspension at a fixed flow rate (1.2 mL/min) through the ADP. The ADP includes a heated capillary, which produces the aerosol of lucinactant at a rate of 36 mg TPL/min (30 mg TPL/mL × 1.2 mL/min). Medical-grade oxygen is supplied to the ADP at ∼3 L/min flow rate, which carries the resultant aerosol concentration of 12 mg TPL/L out of the ADP for delivery.

### Lucinactant radiolabeling procedure

Technetium-99m (^99m^Tc) as sodium pertechnetate was obtained in ∼1.0 mL of solution in normal saline from a clinical pharmacy (Cardinal Health, Inc., Albuquerque, NM). ^99m^Tc was used to prepare a sulfur colloid solution. The sulfur colloid solution was prepared as indicated in the Sulfur Colloid Kit (Pharmalucence, Bedford, MA) with ^99m^Tc solution. When preparing the final radiolabeled formulation for aerosolization, a total of three vials of lucinactant were reconstituted. Two vials were reconstituted with 10 mL of WFI and a third vial was reconstituted with 7 mL of WFI and 3 mL of ^99m^Tc-sulfur colloid solution. The three vials were then combined resulting in a final lucinactant concentration of 30 mg TPL/mL. Typical starting radioactivity of ^99m^Tc was ∼100 mCi, and 50%–65% of the starting radioactivity was transferred to the ADS syringe for aerosol generation. The radioactivity in the ADS syringe was measured using a radioisotope calibrator (Model CRC-12; Capintec Instruments, Inc., Ramsey, NJ) before and after each use.

### Radiolabeling validation procedure

The resultant radiolabeled formulation was aerosolized using the ADS. Particle size samples were collected with a Mercer cascade impactor (In-Tox Products, Moriarty, NM), and wet impactor stages were analyzed for radioactivity. The radioactivity levels were measured by using a radioisotope calibrator. After the impactor stages and backup filter had dried, the stages were analyzed by differential weight (typically after 2–3 days).

The above radiolabeling validation procedure was repeated multiple times (six replicates). The radioactivity and lucinactant mass collected at each Mercer impactor stage/backup filter for all replicates were averaged as presented in the Results section. Using the radioactivity and mass of test article collected on each stage/backup filter of the impactor, the particle size distribution of the aerosol (activity median aerodynamic diameter [AMAD]/mass median aerodynamic diameter [MMAD] and geometric standard deviation [GSD]) was determined using SigmaPlot software (Systat Software, Inc., San Jose, CA).

### Acceptance of lucinactant radiolabeling method

Various criteria for acceptance of the radiolabeling method are available. Data were reviewed against the criteria recommended by the European Medicines Agency (EMA) and the International Society for Aerosols in Medicine (ISAM)/the International Pharmaceutical Consortium on Regulation and Science (IPAC-RS).^(22)^ A similar criterion was also recommended by Devadason et al.^(23)^ These criteria are summarized as follows:

1. The mean ratio of the delivered radiolabeled formulation to the delivered reference formulation should be within 0.85–1.18.
2. The mean ratio of the fine particle fraction of the radiotracer and of the radiolabeled drug to the reference drug should be within 0.85–1.18.
3. If regional lung deposition is to be quantified (e.g., inner and outer lung regions), then the mean ratio of the radiotracer per impactor stage, or group of impactor stages, to the reference drug should be within 0.85–1.18.
4. If regional lung deposition is to be quantified, the mean ratio of radiolabeled drug per impactor stage, or group of impactor stages, to that of the reference drug should be within 0.85–1.18.

The aerosol size distribution of the reference formulation, radiolabeled formulation, and radiotracer was also compared.

### NHP imaging

#### NHP anesthesia

All *in vivo* procedures were conducted at Lovelace Biomedical under protocols approved by the Lovelace Institutional Animal Care and Use Committee. Lovelace facilities are accredited by the Association for Assessment and Accreditation of Laboratory Animal Care International. Lovelace colony animals (*n* = 3) were utilized (2–5 years old, weight range 4.8–6.8 kg) and were returned to the Lovelace colony upon completion of this study. Animals were initially anesthetized with ketamine [5–10 mg/kg, intramuscular (IM)] and transferred to the single-photon emission computed tomography (SPECT) exposure laboratory. Once inside the laboratory, they were placed on intravenous propofol anesthesia, consisting of a bolus loading dose of 2.5–5.0 mg/kg with a 0.2–0.4 mg/(kg·min) maintenance dose. While under anesthesia, animals were continuously monitored for respiratory rate, heart rate, oxygen saturation level, body temperature, and clinical signs. Following aerosol exposure and imaging, animals were allowed to recover from anesthesia. No adverse events were noticed during any procedure associated with this study.

#### Radiolabeled lucinactant aerosol delivery to NHPs

This study consisted of two arms, each with a different setup for the delivery of aerosol to the NHPs. During each arm, three NHPs were delivered radiolabeled lucinactant and imaged. The NHP exposures were performed with anesthetized NHP in the prone position located inside a certified fume hood. The two different delivery setups used during this study are described below.

##### Aerosol delivery circuit

**FIG. 1.**
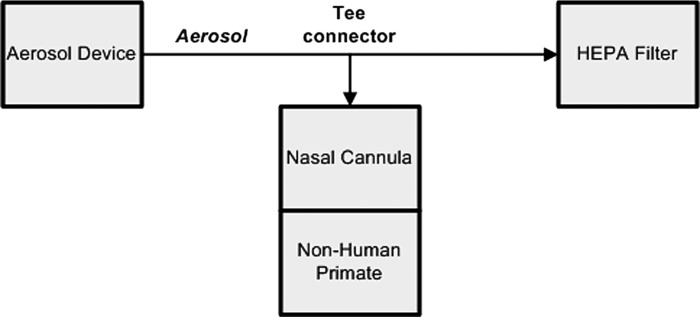
Schematic of the aerosol circuit delivery configuration.

A T-connector was placed within the aerosol delivery tubing, between the ADS and a HEPA filter (Medi/Nuclear Corporation, Inc., Baldwin Park, CA) (Fig. 1). The side arm of the T-connector was connected to a nasal cannula (Part No. BC-4030; Fisher & Paykel HealthCare, Inc., Irvine, CA) for aerosol delivery to the NHPs. Aerosol was pulled continuously through the HEPA filter at a known fixed flow rate (∼2.5 L/min). The NHPs mouth in this setup was closed using medicinal flexible tape while still keeping the sensor for pulse oximeter on the NHPs lip. This resulted in some minor leakage of aerosol from the mouth of the NHPs.

##### nCPAP delivery circuit

**FIG. 2.**
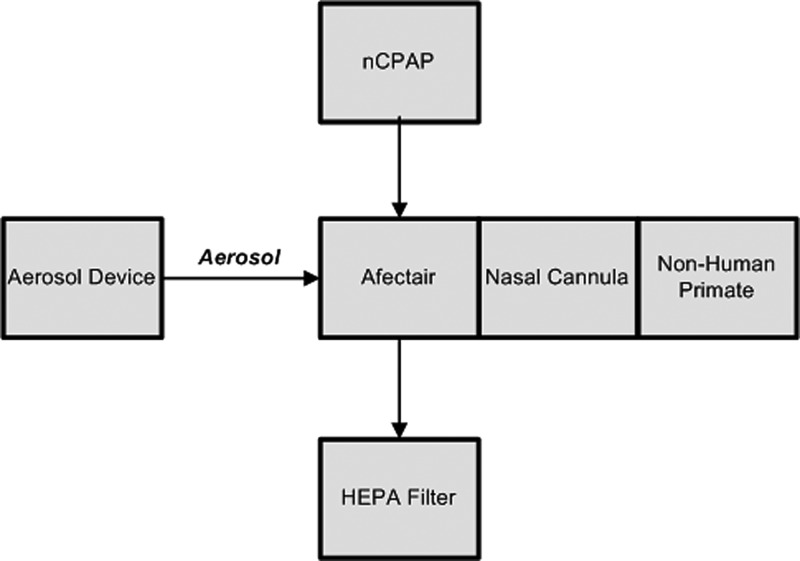
Schematic of the nCPAP circuit delivery configuration. nCPAP, nasal continuous positive airway pressure.

Aerosol was introduced into an nCPAP circuit using an Afectair^®^ device (Afectair Aerosol Conductor, Part No. 809-035, Rev. 01; Windtree Therapeutics, Inc.) (Fig. 2). Afectair replaced the standard Wye connector within the circuit and is designed to shield the aerosol flow from the CPAP flow via a separate aerosol port and a central aerosol channel, thereby preventing dilution of the aerosol.^(24)^

CPAP flow (3 L/min oxygen) was provided to the inflow port of Afectair after conditioning for temperature and humidity using a humidity–temperature controller (Respiratory Humidifier, Part No. MR850JHU; Fisher and Paykel Healthcare Ltd., New Zealand). The temperature–humidity controller was set to invasive mode during the exposure. This setup ensured that the temperature of the aerosol from the ADP did not drop traveling through central channel. The CPAP flow exited Afectair by way of the outflow port, which was connected to the HEPA filter and then to a Bubble CPAP Generator (Part No. BC 153-10, Lot No. 120210; Fisher and Paykel Healthcare Ltd.). The Bubble CPAP Generator was adjusted to 5 cm H_2_O pressure during the exposure.

The aerosol tubing of the ADS was connected to the aerosol port of Afectair. The nasal cannula (Part No. BC-4030; Fisher & Paykel HealthCare, Inc.) was connected to patient interface port of Afectair, directly opposite to the aerosol inlet, using a Bubble CPAP Aerosol Adapter (Part No. BC-185W-140; Fisher and Paykel Healthcare Ltd.).

The NHPs mouth in this setup was closed using medicinal flexible tape while still keeping the sensor for pulse oximeter on the NHPs lip. This resulted in some minor leakage of aerosol from the mouth of the NHPs.

#### NHP imaging

Each NHP was moved immediately to the gamma camera (Siemens ECAM; Siemens Medical Solutions USA, Inc., Malvern, PA) bed to start imaging as soon as possible after the end of radiolabeled lucinactant exposure. Typically, the imaging started within 5 minutes after the end of the exposure. Before the animal was removed from the exposure area for imaging, outside skin of NHP's face was wiped with RadiacWash (Biodex Medical Systems, Inc., Shirley, NY) to remove any radioactivity deposited on the outside surfaces. Individual animals were positioned prone on the ECAM dual-headed gamma camera bed and both gamma camera detectors were accordingly positioned to acquire anterior and posterior images of the animal. The low-energy high-resolution collimators were installed on both detectors. The matrix size and zoom factor were 128 × 128 and 1.0, respectively. A 45- to 90-second planar image (depending on the amount of radioactivity being measured) was acquired for each animal. After the first planar image, a SPECT image was captured that involved rotating the detectors at 16 positions (32 views on a dual-head camera) with each image acquired for a duration of 17 seconds. After completion of the SPECT image, another planar image was acquired for the same duration as the first planar image. The two planar images were used to make sure that the radioactivity and its distribution have not changed significantly due to the biological clearance and radiological decay during SPECT imaging. Region of interest (ROI) analysis was conducted on the lungs, nose + mouth, trachea, and stomach to determine the deposition in each region. Radioactivity was quantified for each NHP individually to determine the radioactivity delivered.

**FIG. 3.**
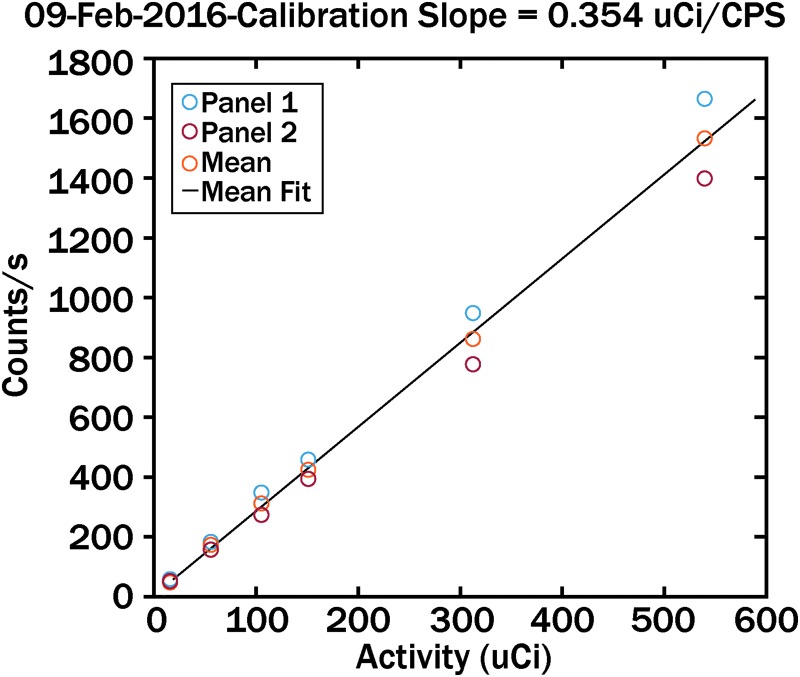
Typical calibration curve correlating radioactivity amounts with gamma camera counts per second.

On each day of exposure, a calibration curve was obtained to correlate known radioactivity with the gamma camera counts. An example calibration curve is shown in Figure 3. The calibration range bracketed the count rate from all analysis data reported.

#### Gamma camera image analysis

Gamma camera image analysis included calibration using the planar calibration data, ROI generation for SPECT and planar images, and generation of an isovolumetric onion skin model. The anterior and posterior images obtained from the gamma camera were averaged and used for analysis. The planar images obtained before and after the SPECT image were also averaged to determine the distribution within the four ROIs. Image reconstruction and analysis were conducted with Matlab R2015b and VivoQuant 2.5. In the onion skin model, the lung was divided into 10 isovolumetric radial shells to quantify the amount of radioactivity deposited in each shell. The shells were numbered 1–10 with 1 being the center most and 10 the outermost shell.^(25)^ The 5 inner most shells (1–5) are combined to represent central deposition, whereas the 5 outer most shells (6–10) are combined to represent peripheral deposition.

**FIG. 4.**
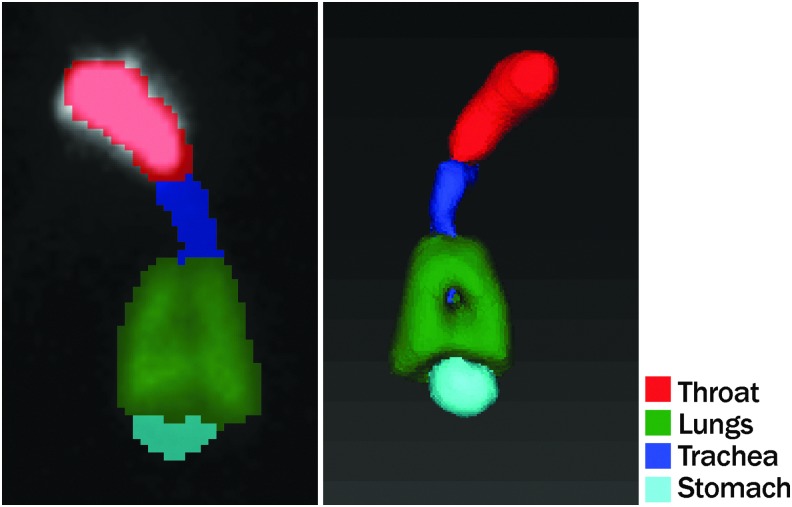
Typical region of interests for planar **(left)** and SPECT **(right)** image analysis. SPECT, single-photon emission computed tomography.

Figure 4 shows typical ROIs used for planar and SPECT images.

#### Experimental conditions

Table 1 details the experimental conditions of different setups used during this study.

**Table 1.** Experimental Parameters Used During This Study

Exposure No.	Setup	Subject ID	NHP weight (kg)	Duration of exposure
1	Aerosol circuit	NHP-1	4.88	7 minutes 17 seconds
2	Aerosol circuit	NHP-2	5.87	3 minutes 0 second
3	Aerosol circuit	NHP-3	6.78	9 minutes 43 seconds
4	nCPAP circuit	NHP-1	4.98	8 minutes 02 seconds
5	nCPAP circuit	NHP-3	6.78	9 minutes 0 second
6	nCPAP circuit	NHP-2	5.87	5 minutes 11 seconds

For the nCPAP delivery circuit, the test order for NHP-2 and NHP-3 was reversed.

nCPAP, nasal continuous positive airway pressure; NHP, nonhuman primate.

#### Calculation of inhaled dose/deposition efficiency

Equation 1 was used to calculate the inhaled dose (ID) from the aerosol radioactivity concentration data. In this calculation, the average radioactivity concentration (microcurrie [μCi]/L) along with actual body weights for NHPs and the duration of exposure were used.


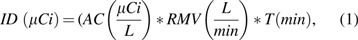


where:

ID = inhaled dose (μCi)

RMV = respiratory minute volume for anesthetized animals = 0.325 × BW^0.789 (26)^

AC = radioactivity aerosol exposure concentration (radioactive analysis, μCi/L)

BW = body weight (kg)

T = duration of exposure (minutes).

Lung deposition efficiency is defined as the ratio of actual deposited radioactivity (determined by gamma image analysis) in the lungs of the NHP to the inhaled dose (Eq. 1). Attenuation factor for these tests was considered 1.0. Equation 2 was used to calculate the lung deposition efficiency.


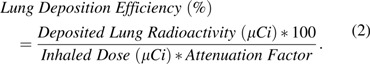


More details about these calculations as they pertain to individual setups are discussed in the Results section.

## Results

### Lucinactant radiolabeling validation

#### Naive mass and radiolabel validation

**FIG. 5.**
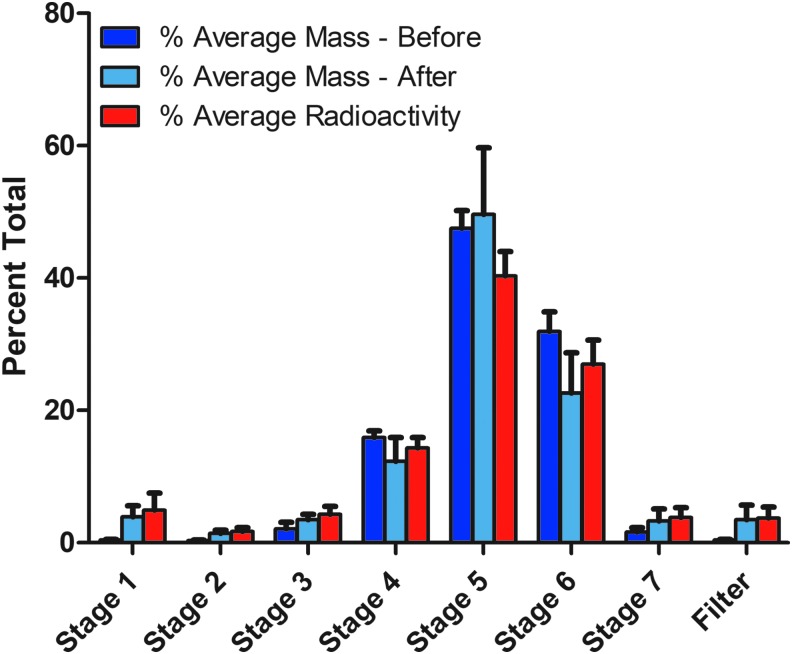
Distribution of mass (before and after radiolabeling) and radioactivity (in % of total).

Table 2 summarizes the % average lucinactant mass-before radiolabeling (naive mass), % average lucinactant mass-after radiolabeling, and % average radioactivity on each Mercer impactor stage and the backup filter. Figure 5 shows comparison among % average lucinactant mass-before radiolabeling, % average lucinactant mass-after radiolabeling, and % average radioactivity. Data are presented with error bars that represent the standard deviation of the percentage for each stage.

**Table 2.** Relationship Between Naive Mass, Radiolabeled Mass, and Radioactivity for Lyophilized Lucinactant Formulation

Stage	% Average mass-before radiolabeling (SD)	% Average mass-after radiolabeling (SD)	% Average radioactivity (SD)
Stage 1	0.4 (0.1)	3.9 (1.7)	4.9 (2.6)
Stage 2	0.3 (0.1)	1.4 (0.5)	1.7 (0.6)
Stage 3	2.1 (1.0)	3.5 (0.8)	4.3 (1.2)
Stage 4	15.9 (1.0)	12.3 (3.6)	14.3 (1.6)
Stage 5	47.5 (2.7)	49.6 (10.1)	40.3 (3.7)
Stage 6	31.9 (3.0)	22.6 (6.1)	27.0 (3.6)
Stage 7	1.6 (0.7)	3.3 (1.8)	3.8 (1.5)
Filter	0.4 (0.1)	3.5 (2.2)	3.7 (1.7)

SD, standard deviation.

It can be seen from Table 2 and Figure 5 that mass of lyophilized lucinactant collected by various stages of the Mercer impactor is similar for the naive tests (mass-before) and the tests with radiolabeled preparation (mass-after). Comparison of mass distribution for the Mercer impactor stages for mass-after and mass-before (naive mass) distributions indicates that the difference in these values is <5.0% for all stages and backup filter except stage 5, which is 7.2%. Radioactivity distribution also follows similar pattern as of naive mass distribution with maximum difference between the radioactivity and mass-before distribution <5.0% except stage 6, where it is 9.3%.

#### Aerodynamic particle size distribution

Table 3 presents a summary of particle size distribution results of all three configurations. It can be seen from Table 3 that both mean AMAD and radiolabeled mean MMAD (mass-after) are within 0.1 μm of the reference mean MMAD (mass-before). Similarly, the mean GSDs between distributions are within 0.3.

**Table 3.** Comparison of Particle Size Distribution

Mass-before radiolabeling		Mass-after radiolabeling		Radioactivity	
MMAD (μm)	GSD	MMAD (μm)	GSD	AMAD (μm)	GSD
2.84 (0.10)	1.78 (0.05)	2.91 (0.24)	1.81 (0.19)	2.92 (0.21)	2.06 (0.16)

Values in parentheses are SD (*n* = 6) of respective values.

AMAD, activity median aerodynamic diameter; GSD, geometric standard deviation; MMAD, mass median aerodynamic diameter.

### Acceptance of lucinactant radiolabeling method

Since this study relates to regional lung deposition, the data were reviewed as it pertains to acceptance criteria 2, 3, and 4 and for particle size distribution.

#### Criterion 2

A comparison of the naive mass (1.00), the radiolabeled mass (1.07), and radioactivity (1.09) within the fine particle fraction region (stage 4 to backup filter) shows that all ratios are within the recommended 0.85–1.18 limit.

#### Criteria 3 and 4

The results of these configurations according to criteria 3 and 4 mentioned above are discussed below:

The results are shown in Table 4. Group 4 is the only group with multiple stages (stages 4, 5, and 6). Both EMA and ISAM recommendations allow use of group of stages to satisfy the recommended criteria. With stages <10% deposition, some stages differed more than ±2% when compared with the reference drug (maximum difference 4.5%, mostly <3.0%). However, this difference does not impact the size distribution in a significant way (as shown in Table 3 earlier). For others, the ratios were within 0.85–1.18 recommended by the guidelines.

**Table 4.** Comparison (Ratio) of Distribution of Mass and Radioactivity of Radiolabeled Aerosol to That of the Reference Drug Aerosol

	Mass-after radiolabeling	Radioactivity
Group 1	3.5%^a^	4.5%^a^
Group 2	1.1%^a^	1.4%^a^
Group 3	1.4%^a^	2.2%^a^
Group 4	0.89	0.90
Group 5	1.7%^a^	0.85
Group 6	3.1%^a^	0.85
Group 7		2.2%^a^
Group 8		3.4%^a^

^a^ For impactor stages with <10% deposits, difference in percent deposition on each stage is reported when compared with percent deposition of reference drug aerosol on respective stages.

These data indicated that the radiolabeling process was validated and appropriate for use in the NHP imaging studies.

### Lucinactant distribution and deposition

The following sections describe the results of the *in vivo* deposition study for each test setup. The results for each setup are divided into three sections. The first section describes the distribution of the radioactivity in the NHPs as measured by planar and SPECT images. The second section describes the calculations for measurement of lung deposition efficiency as they relate to each setup. The third section describes the results of the onion skin model analysis of regional deposition of radioactivity in the lung.

#### Distribution of radioactivity for two aerosol delivery setups

##### Aerosol circuit delivery

**FIG. 6.**
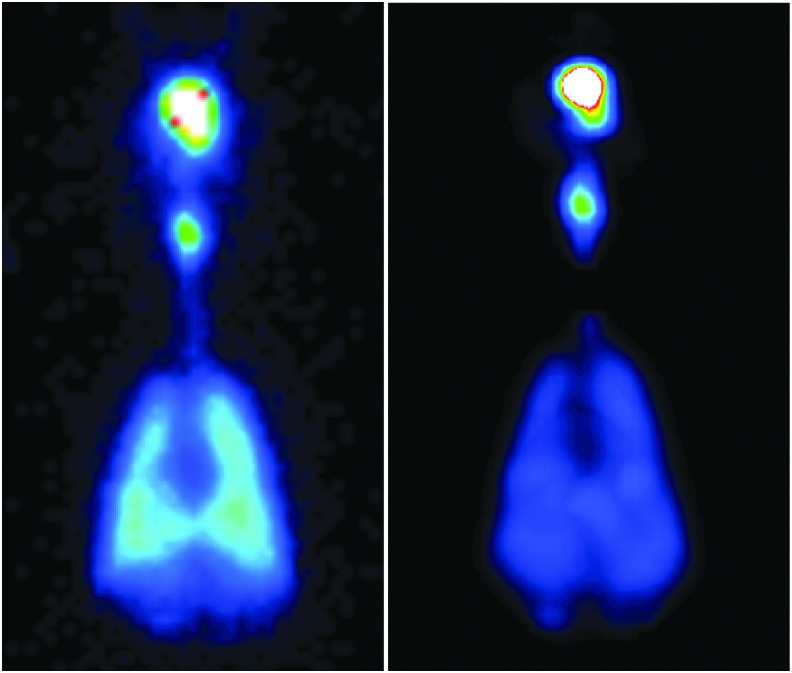
Planar **(left)** and SPECT **(right)** images from NHP-3 for the aerosol circuit delivery. NHP, nonhuman primate.

Figure 6 shows example planar and SPECT images from NHP-3 for the aerosol circuit delivery.

Table 5 shows the distribution of the radioactivity in the four ROIs as measured by planar images. Comparison of individual planar images showed no significant difference in radioactivity and its distribution between planar images before and after the SPECT imaging. Table 5 also shows the distribution of the radioactivity in these four ROI as measured by SPECT images. There is some minor difference between the distributions as measured by two different measurements. However, SPECT measurements are relatively more accurate compared with the planar image measurements in terms of regional analysis. Using SPECT measurement, the ROIs can be better defined, especially when the ROIs overlap. In case of planar images, overlapping ROIs cannot be distinguished properly, which may affect the analysis of the distribution.

**Table 5.** Distribution of Radioactivity in Nonhuman Primates: Aerosol Circuit Delivery

	Nose + mouth (%)	Lungs (%)	Trachea (%)	Stomach (%)
Planar image analysis				
NHP-1	33.85	58.38	7.05	0.72
NHP-2	29.87	66.07	3.27	0.78
NHP-3	39.11	55.89	4.87	0.13
Average	34.28	60.11	5.07	0.54
SD	4.64	5.31	1.90	0.36
SPECT image analysis				
NHP-1	39.77	54.72	5.14	0.37
NHP-2	34.32	61.25	4.09	0.34
NHP-3	46.11	50.11	3.73	0.05
Average	40.07	55.36	4.32	0.25
SD	5.90	5.60	0.73	0.18

SPECT, single-photon emission computed tomography.

Based on SPECT measurements, an average 55.36% ± 5.60% radioactivity was deposited in the lungs of NHPs as a percent of total deposition (range 50.11%–61.25%). It should be noted that on average ∼40% of radioactivity was deposited in the nose and mouth regions, 4% in the trachea, and <0.5% in the stomach.

During image analysis, the number of counts outside these ROIs was measured and characterized as background measurements. Background measurements were not included for the calculations of distribution.

##### nCPAP circuit delivery

**FIG. 7.**
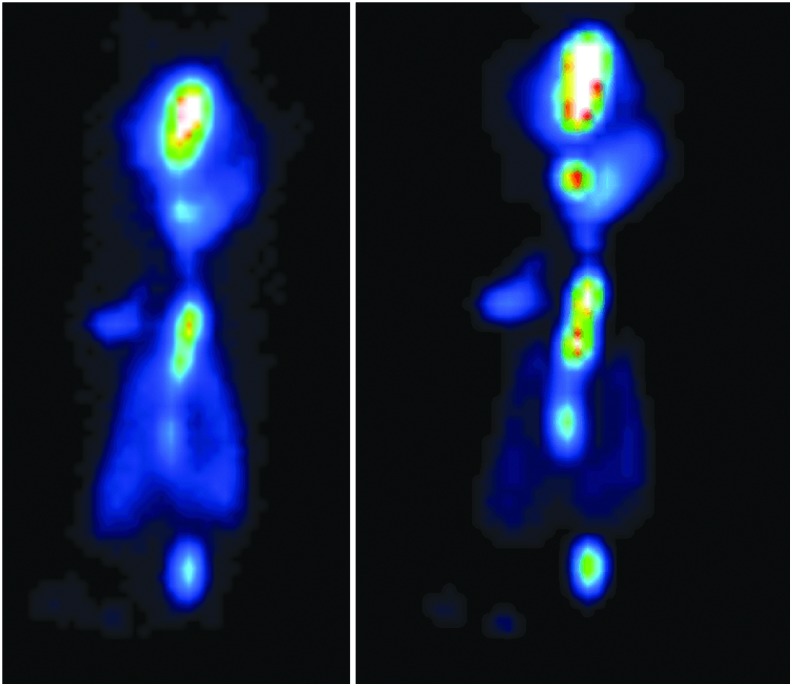
Planar **(left)** and SPECT **(right)** images from NHP-1 for the nCPAP circuit delivery.

Figure 7 shows example planar and SPECT images from NHP-1 for the nCPAP circuit delivery.

Table 6 shows the distribution of the radioactivity in the four ROIs as measured by planar images. Comparison of individual planar images showed no significant difference in radioactivity and its distribution between planar images before and after the SPECT imaging. Table 6 also shows the distribution of the radioactivity in these four ROI as measured by SPECT images.

**Table 6.** Distribution of Radioactivity in Nonhuman Primates: Nasal Continuous Positive Airway Pressure Circuit Delivery

	Nose + mouth (%)	Lungs (%)	Trachea (%)	Stomach (%)
Planar image analysis				
NHP-1	54.44	22.54	18.99	4.03
NHP-2	92.38	5.48	2.06	0.09
NHP-3	92.91	6.22	0.78	0.09
Average	79.91	11.42	7.27	1.40
SD	22.06	9.64	10.16	2.28
SPECT image analysis				
NHP-1	64.72	7.52	24.84	2.91
NHP-2	96.89	2.81	0.29	0.01
NHP-3	97.43	2.46	0.11	0.00
Average	86.35	4.26	8.41	0.98
SD	18.73	2.83	14.23	1.68

SPECT, single-photon emission computed tomography.

The SPECT image from NHP-1 demonstrated large amounts of radioactivity in the trachea (24.84%) and stomach (2.91%). It was noted that anesthesia was lighter in this animal during the exposure and that oral secretions were being swallowed at the end of the test. The high percentage in the stomach may have been due to these swallowed oral deposits. The high percentage in the trachea may include any radioactivity in the esophagus, which cannot be resolved separately from the trachea during image analysis. It is also possible that mucocilliary clearance contributed to the higher observed tracheal deposition. The percentage of radioactivity deposited in the lungs for NHP-1 was 7.52%.

Based on SPECT measurements, >95% radioactivity was deposited in the mouth + nose regions of NHP-2 and NHP-3 with <3% deposited in the lungs as a percent of total deposition. For these two NHPs, negligible amounts of radioactivity were deposited in the stomach and trachea. Slightly higher delivery pressure and flow due to the nCPAP setup resulted in leakage/bypass flow through the mouth/oral cavity. A large amount of radioactivity was deposited in this region as aerosol passed through the oral cavity.

For NHP-2 and NHP-3, there are some minor difference between the distributions as measured by two different measurements.

#### Deposition efficiency for two aerosol delivery setups

The lung deposition efficiency of aerosolized lyophilized lucinactant can be calculated using Equation 2 described in the Methods section.

##### Aerosol circuit delivery

The inhaled dose can be calculated using exposure duration, animal weight, and radioactivity concentration (μCi/L) (Methods section, Eq. 1). For the aerosol circuit delivery, this radioactivity concentration (μCi/L) was calculated using the amount of radioactivity deposited in the exhaust HEPA filter and flow rate maintained through it.

Using these numbers and amounts of radioactivity measured in the NHP lungs, the deposition efficiency was calculated (Methods section, Eq. 2). Table 7 shows the measured amount of radioactivity in various regions of the NHPs for the aerosol circuit delivery, as measured by planar images. It also shows the calculated deposition efficiency for the three NHPs. Average lung deposition efficiency was 6.64% (range 3.33%–11.01%).

**Table 7.** Radioactivity Measured in Various Regions of the Nonhuman Primates and Lung Deposition Efficiency

	Nose + mouth total (μCi)	Lungs total (μCi)	Trachea total (μCi)	Stomach total (μCi)	Total deposited activity (μCi)	Inhaled dose (μCi)	Lung deposition efficiency (%)
Aerosol circuit delivery—planar images							
NHP-1	230.66	396.91	47.51	4.89	679.96	3930.30	11.01
NHP-2	22.02	48.63	2.38	0.58	73.60	1516.77	3.33
NHP-3	137.80	196.84	17.12	0.45	352.21	3669.93	5.58
nCPAP circuit delivery—planar images							
NHP-1	1058.22	437.86	368.12	78.27	1942.47	3289.74	14.07
NHP-2	2713.32	161.00	60.37	2.55	2937.23	4692.67	3.53
NHP-3	1545.95	103.56	12.79	1.43	1663.73	2171.98	4.94

It should be noted that the difference in the actual deposited radioactivity in three NHPs is partly due to different exposure durations and due to slight difference in starting radioactivity in the delivery syringe. While calculating the lung deposition efficiencies, the radioactivity was corrected to account for the radioactive decay based on half-life of ^99m^Tc.

##### nCPAP circuit delivery

Table 7 shows the measured amount of radioactivity in various regions of the NHPs for nCPAP circuit delivery, as measured by planar images. To calculate the lung deposition efficiency for nCPAP circuit delivery, the average delivery efficiency (42.2%) measured for the aerosol circuit delivery was used. Average lung deposition efficiency calculated for the nCPAP circuit delivery NHPs was 7.52% (range 3.53%–14.07%). It should be noted that the difference in the actual deposited radioactivity in three NHPs is partly due to different exposure durations and partly due to slight difference in starting radioactivity in the delivery syringe. While calculating the lung deposition efficiencies, the radioactivity was corrected to account for the radioactive decay based on half-life of ^99m^Tc.

#### Regional lung deposition (onion skin model analysis)

The following section describes the results for the onion skin model analysis of the regional deposition of radioactivity in the lungs.

##### Aerosol delivery circuit

Table 8 shows the results of the percent deposition in 10 isovolumetric radial shells of the lungs of the three NHPs for the aerosol delivery circuit. The percent deposition ranged from 3.6% (shell 10) to 14.1% (shell 5) in this setup with 1 being the central most shell. Aerosolized lucinactant was deposited in both the central (49.5% ± 1.8%) and peripheral (50.5% ± 1.8%) regions of the lungs.

**Table 8.** Percent Deposited in 10 Isovolumetric Radial Shells

Shell No.	NHP-1	NHP-2	NHP-3
Aerosol delivery circuit			
1.	9.7	10.0	10.9
2.	9.4	8.0	7.7
3.	8.5	6.9	8.2
4.	9.1	10.3	10.7
5.	11.7	13.4	14.1
6.	13.0	13.3	13.9
7.	12.5	12.7	12.1
8	10.6	10.9	10.6
9.	9.0	9.1	8.2
10.	6.4	5.4	3.6
nCPAP delivery circuit			
1.	12.9	12.8	11.9
2.	10.6	12.3	9.8
3.	9.5	9.7	7.4
4.	9.0	9.0	9.9
5.	9.7	10.2	13.5
6.	12.3	11.3	14.2
7.	12.8	10.8	11.7
8	10.8	9.7	9.2
9.	8.1	9.2	7.6
10.	4.4	5.1	4.7

##### nCPAP circuit delivery

Table 8 shows the results of the percent deposition in 10 isovolumetric radial shells of the lungs of the three NHPs for the nCPAP delivery circuit. The percent deposition ranged from 4.4% (shell 10) to 14.2% (shell 6) in this setup with 1 being the center most shell. Aerosolized lucinactant was deposited in both the central (52.8% ± 1.2%) and peripheral (47.2% ± 1.2%) regions of the lungs.

## Discussion

Reconstituted lyophilized lucinactant can be aerosolized and delivered noninvasively using the ADS, with and without nCPAP. The aerosol was deposited in the lungs, nose + mouth, trachea, and stomach of NHPs using both delivery methods.

Pulmonary and extrapulmonary distribution varied when aerosolized lucinactant was delivered using an aerosol circuit and an nCPAP circuit. Deposition within the nose + mouth was high with aerosol delivery via nCPAP and was much lower with aerosol delivery via the aerosol circuit. The aerosol circuit delivery resulted in the highest deposition of radioactivity with the lungs, with less deposition within the nose + mouth. Neither of the two setups resulted in appreciable deposition within either the trachea or the stomach. While the distribution of radioactivity within the four ROIs varied, the lung deposition efficiency was similar between the two delivery setups.

The aerosol delivery circuit was designed so that the NHPs inhaled the aerosolized lucinactant through nasal prongs attached to the side arm of a T-connector inserted in the aerosol delivery tubing of the ADS. The amount of radiolabeled aerosol that was deposited within the NHP was a direct function of its minute ventilation. The pattern of deposition (% of the total radiolabel inhaled observed in each of the four ROIs) therefore reflected the quantity of aerosol deposited within the four regions due to the NHPs' respiratory efforts. The efficiency of lung deposition reflects the amount of aerosol that is actually inhaled.

By comparison, the nCPAP delivery circuit has two flow sources: the flow necessary to create and maintain nCPAP and the aerosol flow. The nCPAP flow enters the Afectair connector via the inflow port and exits via the outflow port to a Bubble CPAP Generator. The aerosol flow is introduced via the Afectair aerosol port. Afectair is designed such that, when the NHPs inhale, they breath primarily aerosolized lucinactant from the central aerosol channel. This in itself would be expected to result in a pattern of aerosol deposition similar to that observed with the aerosol delivery circuit. However, the co-oximeter probe attached to the NHPs lip, to monitor their oxygen saturation, caused a small flow leak throughout the respiratory cycle. This leak would cause the nCPAP flow toward the NHP to maintain the set level of pressure. The nCPAP flow would entrain radiolabeled aerosol, thereby increasing the total amount of aerosol deposited, primarily in the nose + mouth during the expiratory phase of the respiratory cycle. As the NHP was not actively inhaling, the excess aerosol would not be deposited in the lungs and the efficiency of lung deposition would be similar to that observed with the aerosol delivery circuit.

The aerosol was observed to be homogeneously deposited in all regions of the lungs. The pattern of regional deposition within the lungs was remarkably similar between the two test setups. Both the central and peripheral deposition of radiolabeled aerosolized lucinactant within the lungs were similar when comparing the two test setups.

To achieve uniform distribution in the lungs, endotracheal surfactant is typically administered rapidly and in relatively large volumes.^(27,28)^ However, rapid intratracheal administration of a large volume of surfactant may cause transient hypoxia, hypercapnia, changes in cerebral blood flow, and intraventricular hemorrhage (IVH).^(29,30)^ This technique may be poorly tolerated by unstable infants. An alternative route of administration is therefore desirable. Nebulized surfactant has been shown to be safer and less harmful compared with intratracheal liquid instillation. A study performed by Dijk et al.^(31)^ has shown that the partial pressure of oxygen in arterial blood increased quickly after instillation compared with a gradual increase during nebulization of Alveofact^®^ over 120 minutes. However, there were also rapid decreases in the mean arterial blood pressure and cerebral blood flow after liquid instillation by 22% and 64%, respectively, whereas there was no change in the mean arterial blood pressure and only 31% gradual drop of cerebral blood flow after nebulization. These findings, as well as a study performed by Perlman et al.,^(32)^ support the hypothesis that nebulized surfactant may decrease the risk of IVH.

Marks et al.^(33)^ showed that both jet and ultrasonic nebulizers could deliver phospholipids extracted from a bovine lung lavage without changing the surface active properties. In a variety of induced lung injury animal models, nebulized surfactant improved both ventilation and lung mechanics, even with minimal deposition in the lungs.^(21,34–37)^ Clinical studies have, however, not always shown a benefit with nebulized surfactant. For example, in a large study, nebulized Exosurf^®^ (colfosceril palmitate) did not improve the outcome of adult patients with acute respiratory distress syndrome (ARDS).^(38)^ These results may be explained by the observation that the delivery of surfactant to the distal lung was insufficient, and the surfactant preparation lacked a protein component that may have affected the ability to lower surface tension, the onset of action of the surfactant, and its susceptibility to serum proteins.

Therapeutic aerosols are administered to ventilated infants commonly with a jet nebulizer. Studies in adults have shown that ∼2.9% of the nebulized dose is deposited in the lungs by this form of nebulization.^(39,40)^ Deposition in ventilated animals and newborns may be even lower, with <2% of the aerosol released into the ventilatory circuit. The rate of deposition to the lungs depends on many factors, for example, placement of the nebulizer in the ventilatory circuit, flow rates used by the aerosol generator, and gas conditions (humidity and temperature).^(41–47)^

Clinical studies for the treatment of neonatal RDS with nebulized surfactant have proven the safety of this approach. However, these studies have been unable to demonstrate consistent effectiveness of this approach. Ventilatory strategies including intermittent MV and nCPAP have been tested with aerosolized delivery of surfactants. Arrøe et al.^(48)^ tested the efficacy and safety of nebulized Exosurf via nCPAP with an estimated deposition to the lung of 10–80 mg per infant. No adverse experiences or improvement in clinical variables were reported as a result of treatment. Berggren et al.^(20)^ treated 34 newborns with RDS using nCPAP and aerosolized Curosurf^®^ (poractant alfa) to test the safety and to determine whether this method of delivery would reduce the need for MV. Curosurf, at a concentration of 20 mg/mL, was aerosolized at rate of 0.2 mL/min, thereby providing a delivered dose to the infant of 4 mg/min. There was no measurement of actual deposition to the lungs. The results did not show superiority of aerosolized surfactant delivery over controls. The study did, however, prove that aerosolized surfactant delivery is safe and did not cause any untoward effects. A pilot feasibility study was performed on preterm newborns with moderate RDS requiring single-prong pharyngeal CPAP for the delivery to the infant of nebulized Alveofact at a dose of 150 mg/kg.^(49)^ The results showed that pharyngeal CPAP alone did not improve ventilation and oxygenation significantly, whereas improvement in oxygenation and alveolar ventilation was noted immediately once nebulization of surfactant was started. The procedure itself was shown to be safe.

Newer technologies may allow the potential for administration of aerosolized surfactants to be deposited in the lungs of premature infants in potentially sufficient mass to effect a therapeutic response similar to that administered by bolus instillation. The Aeroneb Pro provides a high-density aerosol output of small particle size surfactant that still retains surface activity after aerosolization. It can deliver, *in vitro*, a dose of ∼0.6 mg/(kg·min) through nasal prongs, using a specially designed interface over a 3-hour period.^(50)^ This output is comparable to the total dose administered by bolus. Noninvasive aerosol delivery should, therefore, be able to deliver adequate amounts of surfactant and avoid the risks associated with endotracheal intubation, and the adverse of events associated with bolus surfactant administration.

In a small pilot study,^(50)^ administration of aerosolized lucinactant via nCPAP was shown to be safe and well tolerated in 17 infants 28–32 weeks of gestational age at risk for RDS. Variability in output rates of the Aeroneb Pro was observed leading to different average dispensed drug volumes per treatment per patient. Transient desaturations were observed during dosing, without bradycardia or hypotension. Mean FiO_2_ was 0.4 at baseline, and 0.32 at 4-hour post-treatment. Endotracheal rescue surfactant was subsequently required in 29.4% of the infants. A diagnosis of RDS was made at 24 hours in 23.5% of the infants, with 11.8% having BPD at 28 days of life. All infants survived. This study suggests that the administration of aerosolized lucinactant via nCPAP may be a feasible alternative to endotracheal administration of surfactant for the prevention of RDS.

### Limitations

Deposition studies, such as this study, cannot be conducted in preterm human neonates due to the need to utilize a radioactive tracer. NHPs are typically viable alternatives. The cynomolgus macaque was chosen due to the similarities in their respiratory tract anatomy and pulmonary function compared with those of humans.^(51–56)^ Although there were differences in age and size (weight) between the macaques utilized and human neonates, the animals were chosen to best match the respiratory anatomy and pulmonary function between the two species, especially with regard to minute ventilation. While a small number of animals were studied with each delivery circuit (*n* = 3), the use of a primate model justifies this limitation.

Analysis of the deposition of the radiolabel may be effected due to biological clearance and radiological decay during longer duration SPECT imaging. The two planar images, before and after the SPECT images, were used to make sure that the radioactivity and its distribution has not changed significantly. Comparison of individual planar images showed no significant difference in radioactivity and its distribution between planar images before and after the SPECT imaging.

## Conclusions

Efforts to aerosolize surfactants in clinical models have been largely unsuccessful to date^(19)^ because of the limited capability of currently available aerosol generators to aerosolize surfactants in the amount needed to achieve a therapeutic benefit. Compared with surfactant administration via endotracheal instillation, surfactant administration via currently available aerosol generators is highly inefficient and dose delivery is limited.^(20)^ In addition, demonstration of surfactant aerosol deposition has been lacking and/or limited.

This study has demonstrated that the ADS can successfully deliver an aerosol of reconstituted lyophilized lucinactant homogeneously to the lungs under nCPAP. Clinical trials, using the ADS, are currently ongoing to examine the safety and effectiveness of aerosolized lucinactant for the treatment of RDS in preterm neonates.^(57)^

## Notes

Reviewed by:

Ariel Berlinski

Timothy Corcoran
